# Supplementary figures and images for: The mycorrhizal fungi of Cymbidium promote the growth of Dendrobiumofficinale by increasing environmental stress tolerance
Source: PeerJ. 2021 Dec 6;9:e12555. doi: 10.7717/peerj.12555 (PMC8656386; doi:10.7717/peerj.12555)

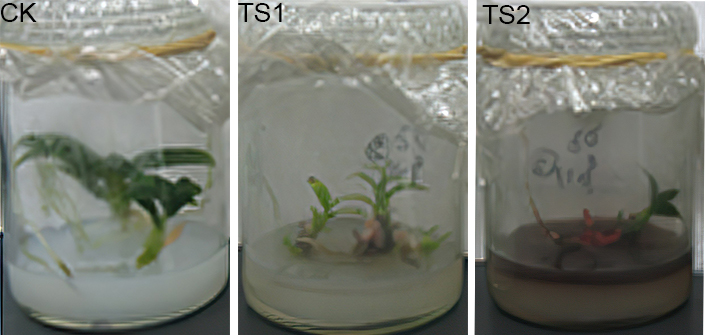

Supplement: Supplemental Information 1 [file peerj-09-12555-s001.jpg]
